# Predicting the resistance of basil entries to downy mildew based on their genetics, pathogen race, growth stage, and environmental conditions

**DOI:** 10.1007/s00425-025-04703-3

**Published:** 2025-05-29

**Authors:** Yariv Ben Naim, Robert Mattera, Yigal Cohen, C. Andrew Wyenandt, James E. Simon

**Affiliations:** 1 New Use Agriculture and Natural Plant Product Program and Department of Plant Biology, Rutgers University, 08901 New Brunswick, NJ US; 2 Faculty of Life Sciences, Bar Ilan University, 5290002 Ramat Gan, Israel

**Keywords:** Basil downy mildew (BDM), Modeling, Oomycetes, *Pb1/Pb2*, *Peronospora belbahrii*, Resistance genes, R genes, Sweet basil

## Abstract

**Main conclusion:**

A model predicting the level of resistance of basil to downy mildew was developed. The model integrates plant age, genetic background, sporulation, disease intensity, pathogen races, and environmental data at an early stage of disease. These results can be used to select and develop new basil cultivars and accelerate the time needed in breeding for basil downy mildew resistance.

**Abstract:**

Basil downy mildew (BDM) caused by the oomycete *Peronospora belbahrii* emerged as a global threat, rapidly becoming the most devastating disease of sweet basil (*Ocimum basilicum*) and other *Ocimum* spp. worldwide. Despite advancements in understanding its biology and epidemiology, and the availability of approved fungicides and management strategies, BDM remains economically destructive and an ongoing risk to basil production worldwide. Recently, the development and introduction of resistant cultivars have emerged as crucial tools in BDM management and the emergence of new BDM races creates new challenges to controlling this disease. The present study aimed to provide growers and breeders with insights into the survival capabilities of resistant basil cultivars under varying genetic backgrounds, pathogen races, growth stages, and various environmental conditions. Through a series of lab and field experiments, we evaluated the response of multiple resistant sources and their lineages to various isolates of *P. belbahrii* across different locations, using multiple indices to assess their resistance. Entries carrying the R genes *Pb1/Pb2* exhibited complete resistance across all races, growth stages, and environmental conditions. Those harboring the R-gene *Pb2* showed similar resistance levels, with minor variability due to growth stage. Responses of *Pb1* plants varied with pathogen race, displaying full resistance to race 0 at all growth stages but displaying susceptibility to race 1. Plant cultivars possessing MRI resistance genes and their recombinant inbred lines (RIL’s) exhibited variable responses to pathogen attacks, ranging from high tolerance to complete susceptibility. Some MRI RIL’s showed high resistance similar to *Pb2* entries. *Pb0* cultivars and 'Eleonora' (unknown background) were susceptible to all races and growth stages in all experiments. Comprehensive analysis across all genetic backgrounds revealed a significant correlation (*R* = 0.73) between disease intensity (D.I) at the seedling stage under controlled conditions and D.I in adult plants under field conditions. Principal Component Analysis (PCA) across six experiments indicated that the primary components influencing disease outcomes were the accession, race, and growth stage, explaining 65%, 22%, and 7% of the variability, respectively. A prediction model based on the statistical parameters residual (%) and root-mean-square error (RMSE) demonstrated strong predictability, particularly regarding pathogen sporulation and daily disease development rates. The model predicted resistance probabilities with *R*^*2*^ values of 0.81, 0.91, and 0.93 at the second, third, and final disease score readings, respectively, significantly earlier (~ 14–21 days post-infection) than traditional assessments (~ 42 days). These findings demonstrate that resistance in basil entries against current pathogen races can be effectively assessed within weeks of disease onset, facilitating more timely and informed management decisions for growers and providing an important tool for plant breeders in search of improved BDM resistance.

## Introduction

Basil downy mildew, caused by the oomycete *Peronospora belbahrii* (Thines), is currently the most devastating foliar disease of sweet basil (*Ocimum basilicum*) worldwide (Wyenandt et al. 2015; Cohen et al. 2017; Simon et al. 2020; Topolovec-Pintarić and Martinko 2020). Despite its global dispersal over 15 years ago, BDM remains one of the most serious pests affecting basil, resulting in severe damage and significant economic losses. Basil is among the most widely consumed aromatic fresh herbs globally (Dudai et al. 2020; McGovern 2023). Strict pesticide residue regulations and zero tolerance for BDM symptoms during postharvest and marketing present significant challenges to commercial growers, limiting effective disease control to a handful of fungicides. Therefore, the primary strategy for managing BDM presence and intensity involves cultivating genetically resistant basil cultivars. Although recent releases of resistant cultivars have offered temporary relief to growers (Simon et al. 2018; Wyenandt 2020), their efficacy is limited as none provide complete protection against the evolving pathogen dynamics.

Research efforts over the past decade have identified highly resistant sources from wild *Ocimum* species, including *O. americanum* var. *pilosum, O. americanum* var*. americanum (*syn. *canum), O. kilimanadascharicum, O. gratissimum, O. campechianum,* and *O. tenuiflorum* (Wyenandt et al. 2010; Farahani-Kofoet et al. 2014; Pyne et al. 2014; Ben Naim et al. 2015a, b). However, integrating these resistance genes into *O. basilicum* remains challenging due to species incompatibilities and polyploidy (Paton and Putievsky 1996; Lekhapan et al. 2021; Matthew et al. 2022). Moderately resistant types, such as *O. basilicum* var. *anisatum, O. basilicum* var. *thyrsiflorum, O. basilicum* var. *citrodorum, O. citrodorum,* and *O. basilicum* var. *minimum,* have shown some durability (Wyenandt et al. 2010; Farahani-Kofoet et al. 2014; Pyne et al. 2014; Ben Naim et al. 2015a, 2015b). However, their complex inheritance mechanisms are not fully understood (Pyne et al. 2018).

Variability in resistance has been observed among different basil cultivars in various geographic locations and under different environmental conditions, possibly due to the presence of different pathogenic races (Ben Naim et al. 2019; Hoffmeister et al. 2020; Ben-Naim and Weitman 2022). Recently, several new BDM-resistant cultivars have been introduced to the market, derived from diverse genetic backgrounds. Cultivars like'Prospera GC-1'and the Prospera collection have demonstrated high resistance in field and greenhouse trials across USA, Europe, and Israel (Cohen et al. 2017). These cultivars often carry the dominant R-gene *Pb1*, derived from *O. americanum* var. *americanum* PI 500945 (Ben Naim et al. 2016; Ben Naim et al. 2018; Cohen and Ben Naim 2019). Additionally, hybrids, such as 'Amazel' (Proven Winners, Carleton, MI, USA) developed through interspecies crosses, have shown high BDM resistance (Brown 2019; Clark et al. 2019; Patel et al. 2021). Other notable resistant cultivars include 'Rutgers Obsession DMR', 'Rutgers Devotion DMR',' Rutgers Passion DMR', and 'Rutgers Thunderstruck DMR', which exhibit varying levels of durability based on the 'MRI' source carrying multiple quantitative trait loci (QTLs) (Silva et al. 2018; Simon et al. 2018; Cooper 2019; McGrath and Sexton 2019; Wyenandt 2020; McGovern 2023). Recent genetic mapping efforts have identified significant QTLs associated with BDM resistance, underscoring the genetic complexity underlying resistance mechanisms (Mattera et al., personal communication). Despite these advancements, some cultivars like 'Eleonora' , 'Loki', and 'Gemini' have shown intermediate resistance, reflecting ongoing challenges in developing universally effective resistant cultivars (Shao and Tian 2018; Brown 2019). The genetic diversity of *P. belbahrii* is increasingly recognized, with studies revealing wide genetic variation among isolates and its known ability to overcome current resistant cultivars in Europe and USA (Ben Naim et al. 2019; Thines et al. 2019; Thines et al. 2020). The introduction of new resistant cultivars to the market may predispose *Ocimum* spp. newly developing pathogenic races, highlighting the need for ongoing research and breeders' vigilance.

In this study, we conducted a series of experiments to evaluate multiple BDM-resistant lines' response to various *P. belbahrii* races at different growth stages and under controlled and open-field conditions across different geographic locations. This comparative analysis aims to provide breeders and growers with insights into the performance of different genetic backgrounds, against diverse pathogen races across different geographic locations, facilitating improved breeding, cultivar selection, and BDM disease management strategies.

## Materials and methods

### Germplasm

A total of 150 entries of *Ocimum* species were comparatively evaluated in the seedling stage and field experiments. This included 8 cultivars carrying *Pb0* (supplied by Johnny’s Seeds, Fairfield, ME, USA; and Neva Yaar, Israel); 1 cultivar with unknown resistance background ‘Eleonora' (EnzaZaden, Enkhuizen, The Netherlands); ‘Mrihani’, ‘Obsession’, ‘Devotion’, and 115 RIL’s of MRI background (supplied by Rutgers University); 15 breeding lines and one commercial cultivar Prospera-F_1_ carrying *Pb1* from PI500945 (supplied by Bar Ilan University); 3 lines carrying *Pb2* from PI500950 (supplied by Bar Ilan University); and 2 lines carrying both *Pb1* and *Pb2* (supplied by Bar Ilan University and Genesis Seeds Ltd, Ashalim, Israel).

### Experimental design

Experiments 1 and 2 were conducted in controlled growth chambers, while Experiments 3, 4, 5, and 6 were performed in the field. Table 1 summarizes the information regarding the nature of the experiments, used races of the pathogen, locations, and the plant growth stage.

**Table 1:** Description of host and environmental conditions prevailing in Experiments 1–6

Exp	Environment	Growth stage	Genetic backgrounds	Race/name	Avg. min temp (°C)	Avg. max temp (°C)	Avg. dew point (°C)	Avg. night RH (%)	% days with ≥ 6 h RH ≥ 95%
1	Growth chamber	Seedling	Pb-0,1,2,1/2, MRI	0 (Knafo 3)	± 24	± 24	18*	60 (100)*	–
2	Growth chamber	Seedling	Pb-0,1,2,1/2, MRI	1((Mop Negev)	± 24	± 24	18*	60 (100)*	–
3	Field	Adult	Pb-0,1,2,1/2, MRI	0 (Knafo 3)	21.1	24.3	18.5	79.9	85%
4	Field	Adult	Pb-0,1,2,1/2, MRI	1 (Mop Negev)	24.7	27.6	21.1	81.2	81%
5	Field	Adult	Pb-0,1,2,1/2, MRI	0 + 1 (Knafo 3 + Mop Negev)	24.9	28.3	19.6	73	63.5%
6	Field	Adult	Pb-0,1, MRI	Unknown	18.8	28.6	16.2	94	48.5%

^*^Average values in dew chamber

In Experiments 1 and 2, basil plants were grown in multicell trays (cell size 2.5 × 2.5 cm) filled with a potting mixture (peat: vermiculite 3:1, v/v), with one plant per cell. Before sowing, seeds were gently scraped with sandpaper (P 320) to improve germination. Upon germination, plants were fertilized with 0.5% 20–20-20 NPK solution twice a week. Plants were grown in a controlled growth chamber with 14 h photoperiod, photon flux density (PPFD, 200 µmol m^−2^ s^−1^) at 24 °C. At the 2–4 true leaf stage (27–30 days after seeding), seedlings were spray-inoculated with 5,000 spores/ml, each tray with a different isolate of *P. belbahrii*. The inoculated plants were incubated in a dew chamber [100% relative humidity (RH), 18 °C, darkness] for 14 h and then returned to the growth chamber for symptom development. At 10 days post-infection (dpi), the infected plants were returned to the dew chamber for 15 h to induce sporulation.

Experiments 3–5 were conducted at Bar Ilan University farm, Israel (32°4′9′′ N, 34°50′35′′ E), and Experiment 6 was conducted at Rutgers University Snyder Research & Extension Farm, Pittstown, New Jersey, USA (40.557594, −74.960232). Planting dates and disease scoring periods for each experiment are detailed in Table 1.

In all field experiments, plants were planted at the 4–6 leaf stage (35–45 days after seeding). The experiments were set with 3 replications, about ~ 8 plants from each accession were planted in each while from the control cultivars ‘Aroma 2’,’SB22’, and ‘Peri’ (*Pb0)*, 15 plants were used in each replication. At planting, all plots were sprayed with 0.1% Ridomil-Gold MZ (mefenoxam 4% + mancozeb 64%) to prevent spontaneous infection with downy mildew. ~ 21 days after the planting (a total of 60 days from seeding) when the plants reached the 8–12 leaf stage, and mefenoxam lost its activity, downy mildew-infected basil plants were planted as spreaders between plots. Four spreader plants were planted in reserved spaces that were kept between six random plots (~ 60 experimental plants) to ensure uniform infection across the field. Those spreader plants, together with control plants, were used to ensure continued infection in the field.

### Pathogen and inoculation

To prevent cross-contamination of isolates in our experiments, sporulating leaves were stored at −80 °C. One week before each experiment, sporulating leaves were taken out from the −80 °C and spores were used to inoculate healthy plants as described below (plants were grown in a controlled greenhouse). The process was repeated for each experiment with its own required isolate. The infected plants were used as a starter for each of the experiments.

Isolate 'Knafo 3'(collected in 2014 at Ein-Tamar, Southern Jordan Valley, Israel), belonging to race 0, was used in Experiments 1, 3, and 5. Isolate 'Mop Negev' (collected in 2014 at Ein-Tamar, Southern Jordan Valley, Israel), belonging to race 1, was used in Experiments 2, 4, and 5. These two isolates exhibit differential virulence profiles (Ben-Naim and Weitman 2022). In Experiment 5, isolate 'Knafo 3' was introduced first with spreader plants, and 10 days after disease appearance, the 'Knafo 3' spreader plants were removed from the soil and 'Mop Negev' spreader plants were planted instead. For all experiments except for Experiment 6, potted plant of ‘Peri’ (3–5 plants, 4–6 leaves) were spray-inoculated with 5,000 spores/ml, with a different isolate of *P. belbahrii*. The inoculated plants were incubated in a dew chamber (100% RH, 18 °C, darkness) for 14 h and then returned to the growth chamber for symptom development. At 10 days post-infection (dpi), the infected plants were planted as a spreader plants between the accessions in each experiment.

In Experiment 6, plants were exposed to natural infection with an unknown race. The extra plants of ‘Aroma 2’, ‘SB22’, and ‘Peri’ and their wide dispersion in field ensure strong natural infection, consistency of the disease, and secondary spores’ dispersions all over the experiments time.

### Disease assessment

Disease assessment was done every 4–5 days at the seedling stage, while assessments of mature plants in the field were done every 7–9 days. The first scoring began immediately upon onset of symptoms.

The following indices were used to assess the response of basil plants to downy mildew infection:

1. Disease intensity (D.I): Each plant was visually scored 5–6 times during the season using a 0–4 scale:

0 = no visible symptoms

1 = 1–10% of the leaves show chlorotic symptoms; lesions occupy 0–10% of the total leaf area

2 = 10–25% of the leaves show chlorotic symptoms; lesions occupy up to 25% of the total leaf area

3 = 25–50% of the leaves show chlorotic symptoms; lesions occupy up to 50% of the total leaf area

4 = 50–100% of the leaves show chlorotic or necrotic symptoms; lesions occupy over 50% of the leaf area, infected leaves desiccate, fall, and plants often show wilt symptoms.

(2) Fungal sporulation: The intensity of sporulation of *P. belbahrii* on infected leaves was visually evaluated using a 1–4 scale:

1 = No sporulation, or negligible sporulation on the edges of chlorotic lesions

2 = Slight sporulation in the center of chlorotic lesions, covering up to 25% of the chlorotic leaf area

3 = Moderate sporulation, covering 50% of the chlorotic leaf area

4 = Abundant sporulation covering 50–100% of the chlorotic leaf area.

(3) Qualitative marketing rating (M.R): Plants were visually classified using a 1–3 scale:

1 = No or few symptoms, acceptable for marketing

2 = Medium disease level, not suitable for marketing although most plants look healthy

3 = Severe symptoms, no market value.

(4) Disease development rate (DDR) This index was used to determine plant resistance by the daily development rate of the disease. At first, the D.I scores taken during the epidemic were used to calculate the Area Under Disease Progress Curve (AUDPC) as described by the following formula:
  $$AUDPC={\sum }_{i=1}^{n-1}\left(\frac{{\text{y}}_{i}+{y}_{i+1} }{2}\right)({t}_{i+1}-{t}_{i}),$$

where y_*i*_ is BDM incidence at the time *i*, *n* is the number of data taken, and *t* is the number of days between the registration of *t*_*i*_ and *t*_*i*+*1*_.

These area under disease progress curve (AUDPC) values were used to calculate the standard area under disease progress Curve (SAUDPC) according to the following formula:

$$SAUDPC = \frac{{AUDPC}}{{Total\,\,trial\,\,days}}*100.$$

The daily disease progress rate (DDR) was calculated by dividing the SAUDPC by the total number of days of an experiment

$$\% DDR = \frac{{SAUDPC}}{{Total\,\,trial\,\,days}}.$$

### Data analysis

All obtained data were analyzed using JMP Pro commercial software tools. Disease intensity and epidemiological variables were subjected to protect the overall error rate with paired Tukey HSD analysis of variance and correlation (Steel and Torrie 1981). The least significant difference (LSD) test was employed to the means separation based on the JMP default (at *P* ≤ 0.05).

The 113 MRI RILs (a total of ~ 7000 plants in the six experiments) showed extreme variation in disease intensity. To minimize the large number of accessions, we used for downstream analysis the Jmp17pro partition algorithm which separate the MRI RIL’s plants into major groups by classification of trees (Partition). The algorithm associated several main groups based on average disease intensity and then by examining each result (Individual). After having the software partition, we manually increased the number of groups while increasing the values of *R*^*2*^. The partition was ultimately determined with maximal *R*^*2*^ while maintaining minimum splits.

### Prediction by actual models

Disease intensity of the six genetic backgrounds was used to develop a prediction model. We used the data obtained in Experiments 1–4 to predict the results that should have been obtained in Experiments 5–6. The following variables were recorded and analyzed: pathogen race, plant genetic background, plant growth stage, disease intensity, fungal sporulation, disease development rate, and normalized disease development rate. The model was used to validate the results of Experiments 1–4 by comparing the regression coefficients (*R*^*2*^*)* yielded by the F test (Harrell 2015, 2017).

The model was evaluated in three steps based on the method described by Chatterjee and Hadi (2015): (i) comparison of physical theory with dependent variables and regression coefficients; (ii) comparison between observed and predicted values; (iii) collection of new data to check predictions. The assessment of predictions was conducted through the root-mean-square error (RMSE) and error percentage using the following formula:

$$RMSE={\left[\frac{\sum_{i=1}^{n}{\left(Pi-0i\right)}^{2}}{n}\right]}^{0.5}$$

$$Error\,percentage\, = \,\left( {Pi - 010i} \right)100$$

*Pi* and *Oi* are the predicted and observed values for the studied variables, respectively, whereas n is the total number of observations.

A predictive model for disease intensity was developed based on the environmental variables by performing stepwise multiple regression analysis (Meyer and Woodroofe 2000). Using the equations below, the coefficient of determination (*R*^*2*^) was calculated. *R*^*2*^Adj. was used to determine the strength of the relationship between individual environmental variables and disease and to test the model’s prediction accuracy (Steel and Torrie 1981).

$$R^{2} = \frac{{Regression\,\,sum\,\,of\,\,square}}{{Total\,\,sum\,\,of\,\,square}} = 1 - \frac{{Error\,\,sum\,\,of\,\,square}}{{Total\,\,sum\,\,of\,\,square}}$$

$${R}_{adj.}^{2}=1-\frac{\left(1-{R}^{2}\right)\left(n-1\right)}{\left(n-k-1\right)},$$

where *n* denotes the sample size, and k is the number of independent variables. Mean square error and Mallows’ Cp were also calculated to evaluate the influence of the independent variables included in the model using the following expressions (Steel and Torrie 1981).

### Principal component analysis (PCA)

All genetic backgrounds were evaluated via PCA by using four indices: D.I, sporulation rate, AUDPC (the standardized AUDPC and DDR were excluded as they are derivatives of AUDPC) and M.R. The final PCA analysis was based on 16 variable combinations which include the 4 disease assessments (D.I, sporulation, M.R, AUDPC) * 2 ages* 2 races.

## Results

### Partition

The partition of the MRI RIL’s D.I in Experiments 1–4 showed an average grouping of 7.5. The maximal partition of 11 group was at seedling stage with race 1, and minimal partition of 4 groups at adult stage with race 1 (Table 2). The partition of the MRI-RIL’s AUDPC of the four Experiments showed an average grouping of 8.5. The maximal partition of 11 group was at seedling stage with race 0, and minimal partition of 7 groups at seedling stage and adult stage with race 1. The grouping by partition results was adjusted by maximal *R*^*2*^ which ranged from average 0.81 to 0.91 with ~ 1350 plants/Experiment. The grouping by partition results was used for Tukey HSD comparison of Experiments 1–4.

**Table 2:** Disease intensity in MRI recombinant isogenic lines (RIL’s) and area under the disease progress curve (AUDPC) partition in Experiments 1–4. For each experiment the number of individuals in each group and the mean (underline) of each group was given

Groups													
Experiment/record	G1	G2	G3	G4	G5	G6	G7	G8	G9	G10	G11	Total *n* =	*R*^*2*^
Race-0 Seedling D. Intensity												1336	0.81
No. individuals:	112	103	118	159	239	304	32	269					
Mean:	5.3	22.2	35.2	50.8	69.8	89.6	95.3	100					
Race-0 Seedling AUDPC												1336	0.82
No. individuals:	97	126	134	83	343	236	86	22	131	32	46		
Mean:	14.4	69.4	147.9	206.4	263	348.4	379.9	392	400	403.4	412.5		
Race-1 Seedling D. Intensity												1333	0.81
No. individuals:	19	32	61	61	44	91	206	214	324	21	260		
Mean:	2.1	5.9	15.2	26.4	32.3	39.9	53.6	71.6	91.7	98.1	100		
Race-1 Seedling AUDPC												1333	0.81
No. individuals:	181	153	119	352	239	108	181						
Mean:	51.2	140.5	205.2	272.1	357.8	393.1	412.1						
Race-0 Adult D. Intensity												988	0.91
No. individuals:	6	43	83	203	241	141	271						
Mean:	12.5	41.9	50.8	65	81.7	90.3	98.4						
Race-0 Adult AUDPC												988	0.91
No. individuals:	21	25	118	162	118	88	196	197	63				
Mean:	262.3	454	621.4	865	1031.7	1206.5	1369.9	1535.3	1764.4				
Race-1 Adult D. Intensity												931	0.9
No. individuals:	45	196	308	382									
Mean:	23.1	50.7	77.3	94.2									
Race-1 Adult AUDPC												932	0.91
No. individuals:	48	118	52	186	129	186	213						
Mean:	676	1600.2	2237.1	2685.3	3121.3	3488.8	3968.4						

### Tukey HSD analysis

**Fig. 1:**
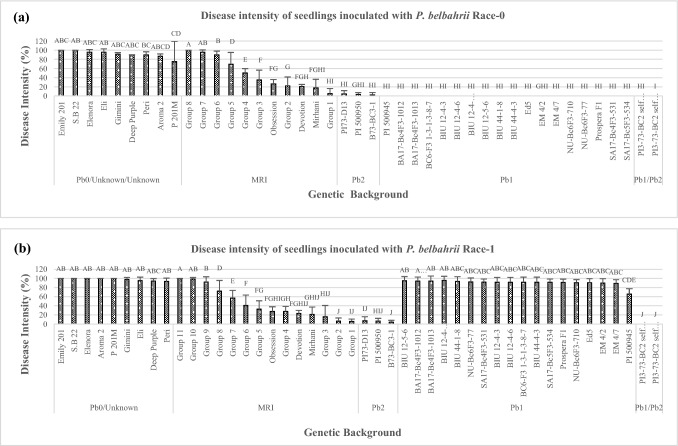
The final scores of the disease intensity of basil seedlings inoculated by two different isolates. **A** Race 0 (*n* = 809). **B** Race 1(*n* = 712). Tukey’s HSD values ± SD (*P* ≤ 0.05). Different letters indicate significant differences

**Fig. 2:**
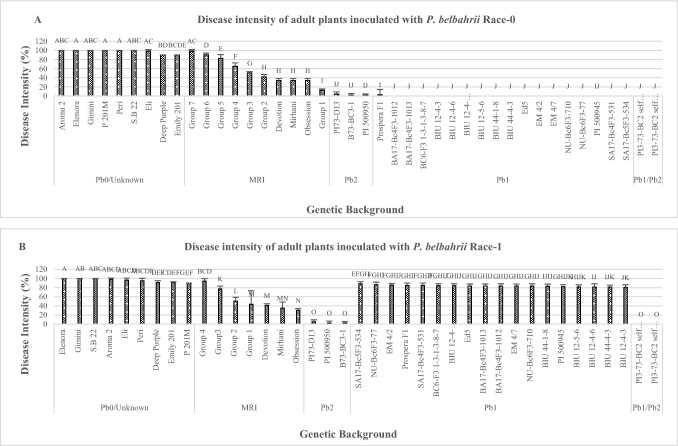
The final scores of the disease intensity of adult basil plants in field inoculated by two different isolates. **A** Race 0 (*n* = 669). **B** Race 1(*n* = 668). Tukey’s HSD values ± SD (*P* ≤ 0.05). Different letters indicate significant differences

Similar D.I levels were observed at the seedlings stage and at adult stage in the field. Seedlings and the adult plants carrying the same genetic backgrounds of both showed similar D.I when subjected to a certain race (Figs. 1A–B and 2A–B). When D.I was measured at the seedling stage using race 0, most genetic backgrounds showed some levels of resistance, whereas all *Pb 0* entries and'Eleonora'were highly susceptible (Fig. 1A). The *Pb1/Pb2* and *Pb1* plants showed complete resistance. The MRI-group 1,'Mrihani', 'Devotion' and *Pb2* lines, showed high incomplete resistance which did not differ statistically from the *Pb1/Pb2* and *Pb1* plants. Similar results were observed in the field Experiments for *Pb1/Pb2*, *Pb, Pb2,* and MRI-group1 (Fig. 2A).

**Fig. 3:**
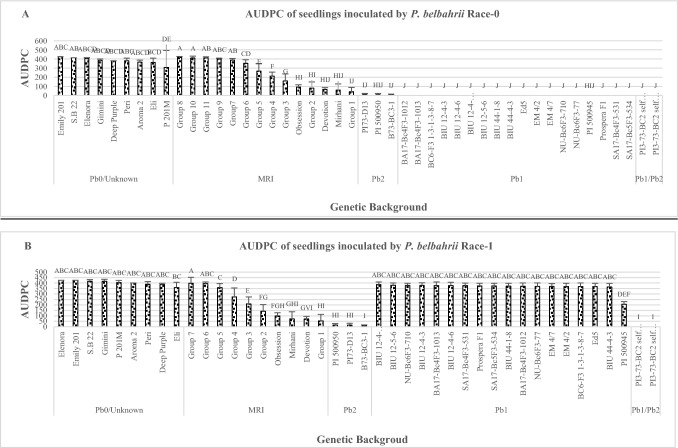
Area under the disease progress curve (AUDPC) of basil seedlings inoculated by two different isolates. **A** Race 0 (*n* = 809). **B** Race 1(*n* = 712). Tukey’s HSD values ± SD (*P* ≤ 0.05). Different letters indicate significant differences

**Fig. 4:**
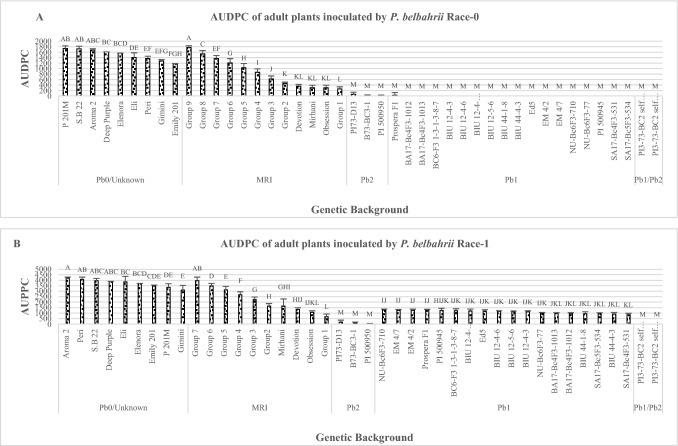
Area under the disease progress curve (AUDPC) of adult basil plants in field inoculated by two different isolates. **A** Race 0 (*n* = 669). **B** Race 1(*n* = 668). Tukey’s HSD values ± SD (*P* ≤ 0.05). Different letters indicate significant differences

When D.I was measured using race 1 at the seedling stage, only *Pb1/Pb2* showed complete resistance without any sign of disease. *Pb2* lines showed high incomplete resistance (limited chlorotic lesions) which did not differ from MRI-groups 1–3,'Mrihani' and 'Devotion' (Fig. 1B). All *Pb1* plants except the wild resistance source PI 500945 were as completely susceptible as *Pb0.* Similar results were observed in the field Experiments (Fig. 2B). The *Pb1/Pb2*, *Pb2* entries were the only resistant plants and were significantly different from backgrounds including the MRI-group AUDPC values were calculated for seedling and adult plants inoculated with race 0 and race 1 (Figs. 3A–B and 4A–B). When race 0 was used, AUDPC values of seedling and adult plants were similar for *Pb1/Pb2*, *Pb2*, *Pb1* MRI-group 1,'Mrihani', and 'Devotion' (Fig. 3A). However, at the adult stage, *Pb1/Pb2*, *Pb2*, and *Pb1* were significantly more resistant than MRI-group 1, 'Mrihani' and 'Devotion' which showed incomplete resistance with limited chlorotic lesions (Fig. 4A).

When race 1 was used, similar AUDPC values were seen for seedling and adult plants with field values being lower. At seedling stage *Pb1/Pb2*, *Pb2*, MRI-group 1, 'Mrihani') and 'Devotion', showed the same levels of resistance, while at adult stage, only *Pb1/Pb2* and *Pb2* were significantly more resist than all other entries, including all MRI entries which showed high incomplete resistance (Figs. 3B and 4B).

While the AUDPC values of seedling and adult plants exposed to race 0 were similar, AUDPC values of seedling and adult plants exposed to race 1 were similar for all genetic backgrounds except to *Pb1* background (Figs. 3A and 4A). The AUDPC values of the adult *Pb1* background were lower than that of the seedling stage (Fig. 4B). It appears that *Pb1* gene is not effective against race 1 but compared with the seedlings Experiment, the *Pb1* gene showed lower rate of disease development throughout the Experiment, and in some cases, it was as effective as the MRI-group 1–2, 'Mrihani', 'Obsession', and 'Devotion'.

D.I and AUDPC were equally suitable to serve as the ultimate means of adequately assessing plant resistance. Both showed consistent evaluation of resistance except for one flaccid case (Figs. 1 and 2 compared with Figs. 3 and 4). The trend of all genetic background’s D.I and AUDPC Experiments with two races was consistent except for the AUDPC of *Pb1* genetic background (Figs. 3B and 4B). The AUDPC of adult plants of *Pb1* genetic background inoculated with race 1 was higher than D.I of seedlings. The lower AUDPC values compared with D.I indicate that disease development rate in the field was low and probably affected by the physiological maturity of the plants or by other factors during the early–middle stages of the experiment.

### Multivariate analyses

The relationships (in Experiments 1–4) between disease values, host genetic background, pathogen race, and plant age were analyzed using principal component analysis (PCA), correlation analyses, and multivariate regression analyses.

**Fig. 5:**
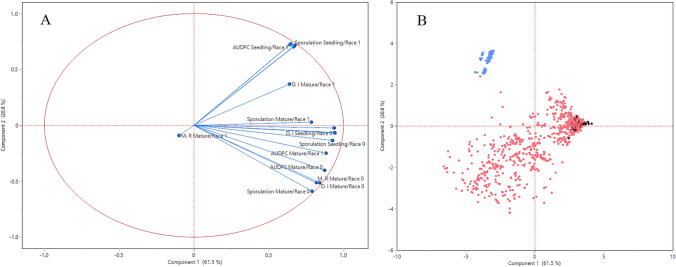
Principal component analysis within Experiments 1–4. **A** Score plot of components 1 and 2. **B** Biplot (scatter plot) of components 1 and 2

**PCA** All genetic backgrounds were evaluated via PCA using four indices: D.I, sporulation rate, AUDPC, and M.R (the standardized AUDPC and DDR were excluded as they are derivatives of AUDPC). The PCA analysis showed that all 16 variables (4 background × 4 indices) converged into two main components which explain 82.3% of the variances, in which component 1 explains 61.5% and component 2 explains 21% (Fig. 5A). The components are also represented by a scatter plot PCA (Fig. 5B). With lack of variance, the minimal variation of the genetic backgrounds *Pb1/Pb2*, *Pb2*, and ‘Eleonora’ is not shown in the scatter plot, while *Pb1* is excluded from *Pb0* and MRI backgrounds indices. For better understanding of the other principles and indices, we examined the number of eigenvalues and their coverage.

**Fig. 6:**
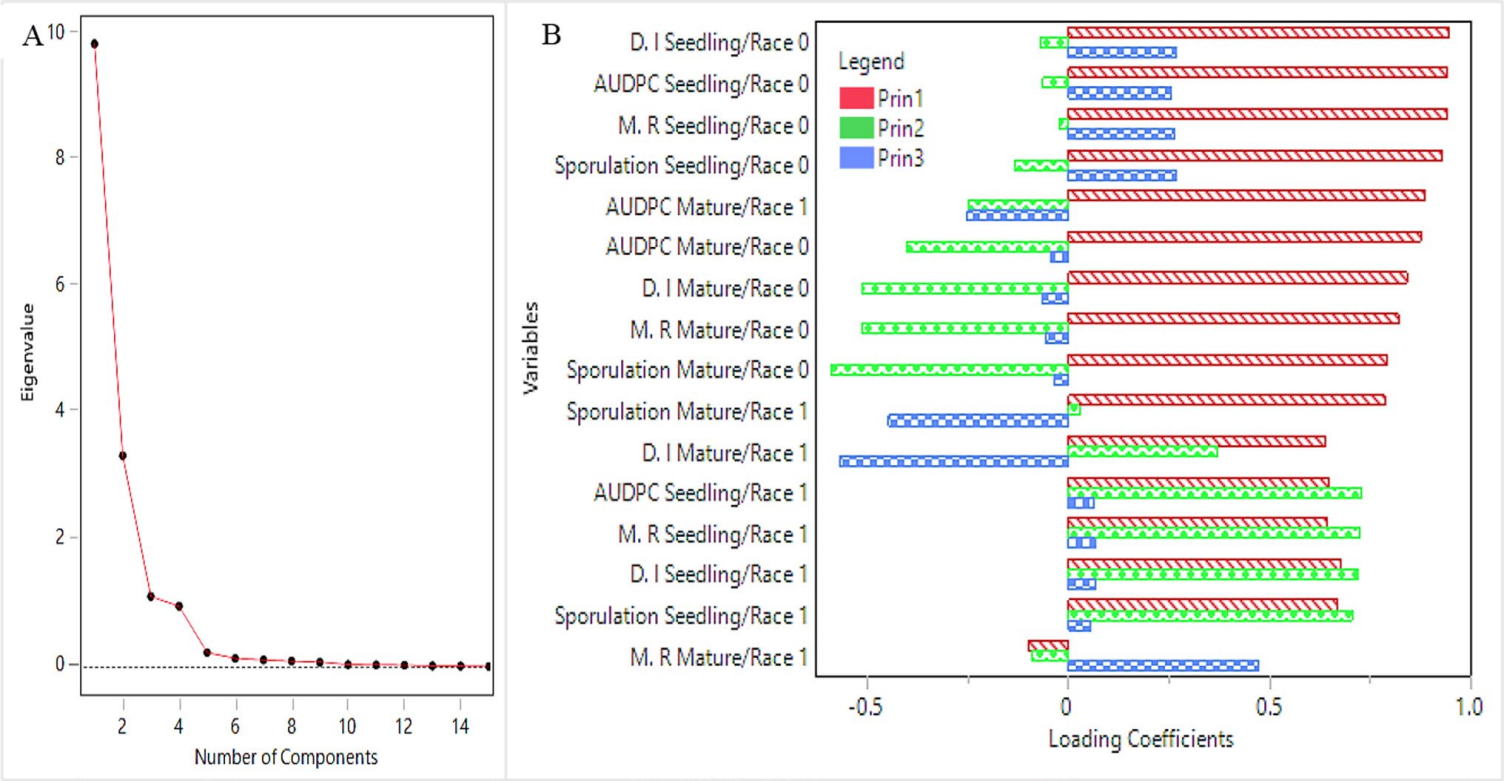
The relation between the components/eigenvalues (**A**) and the loading coefficients of the four disease indices with the three highest principals (**B**)

Out of the 16 variables, 7 eigenvalues were found by the PCA analysis (Table 3) in which the eigenvalues 1–4 were 9.8, 3.3, 1.1, and 0.95 with components % of 61.5, 20.8, 6.9, and 5.98, respectively. The total components percent of the four eigenvalues stands at 95.2 (ChiSq Prob < 0.0001). The Screen plot shows that the main variance distribution can jointly explained by 3–4 components as represented by the eigenvalues (Fig. 6A). For better understanding of the growth stage and race effects with the principles, we used the loading coefficients analysis with the three highest principles that were obtained from the Screen plot. The three principal component loading coefficients analysis shown in Fig. 6B indicate that principal component 1 explains the positively shared variance with all variables (except for one offside, Marketing Rate/adult/Race1) as can be seen in the red bars. In contrast, principal component 2 is affected mainly by the race (green bars) in which race 1 is mainly positively affected, while race 0 is negatively affected. The positive trend is reserved for all indices (except for the “AUDPC/Adult Race 1”). The third principle (blue bars) is associated with age, in which seedlings are positively affected, while adult plants are negatively affected. This trend is true for both races and all indices.

**Table 3:** List of eigenvalues and their cumulative (cum.) percent

Number	Eigenvalue	Percent	Cum. percent	Chi-square	DF	*P* > ChiSq
1	9.843355	61.515	61.515	58,230.4	112.007	<.0001
2	3.335337	20.844	82.359	37,763.8	114.443	<.0001
3	1.110302	6.939	89.297	23,237.4	105.059	<.0001
4	0.957896	5.986	95.283	16,164.3	92.043	<.0001
5	0.225052	1.406	96.690	3447.54	79.283	<.0001
6	0.132563	0.828	97.518	378.814	67.208	<.0001
7	0.108193	0.676	98.194	0.000	55.941	1.0000

### Correlation

Following the PCA results, we examined the variables and indices for their best association with D.I by correlation analysis. The statistical analysis was done for each major genetic background (*Pb* and MRI) separately and for both together.

A correlation of 0.97 and 0.94 was calculated for race 0 and race 1, respectively, when the genetic backgrounds of *Pb* genes were examined between the seedling stage and the adult stage (Table 4). These similar correlation values indicate that D.I is not affected by plant age. When MRI-RILs were tested, the correlation was 0.52 for both races, indicating a moderate correlation between D.I in the seedling stage and the adult stage without effect of the race. When the correlation of all genetic backgrounds between the seedling stage and the adult stage of the two races was examined, the correlation was 0.8 for race 0 and 0.66 for race 1, indicating a weaker association between D.I and plant age.

**Table 4:** Correlation between disease intensity and pathogen race, plant age, and plant genetic background

	Impact factor		*Pb* background		MRI background		All background		
Power	Race	Growth Stage	Correlation	Counts	Correlation	Counts	Correlation	Counts	*P*
By races	0	Seedling/adult plant	0.9676	475	0.5265	1003	0.801	1478	<.0001
	1	Seedling/adult plant	0.9438	433	0.5173	947	0.6583	1380	<.0001
By age	0/1	Seedling	0.3109	488	0.9470	1363	0.6078	1851	<.0001
	0/1	Adult plant	0.3972	431	0.7314	848	0.3976	1279	<.0001
By race and Age	0/1	Seedling/adult plant	0.3838	433	0.5269	947	0.3979	1380	<.0001
	1/0	Seedling/adult plant	0.3145	475	0.5537	1003	0.2049	1478	<.0001

**Fig. 7:**
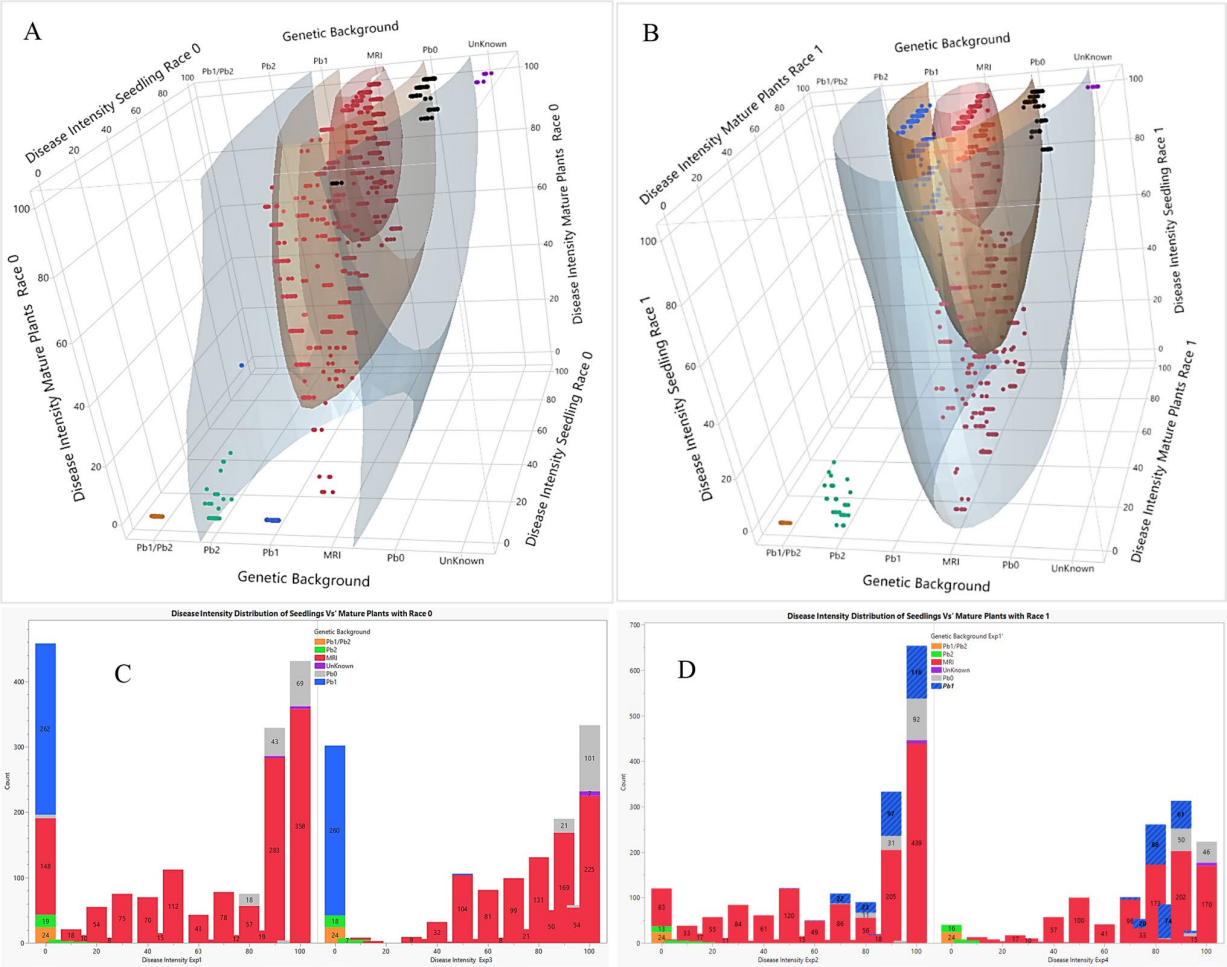
**A-B** 3D-scatter plots of the disease intensity (D.I) of the genetic backgrounds at seedling and adult stages inoculated by race 0 and race 1 isolates. **C**-**D** The D.I distribution histograms of seedlings and adult plants inoculated with race 0 and race 1. **A** and **C** *n* = 1478; **B** and **D** *n* = 1380

**Fig. 8:**
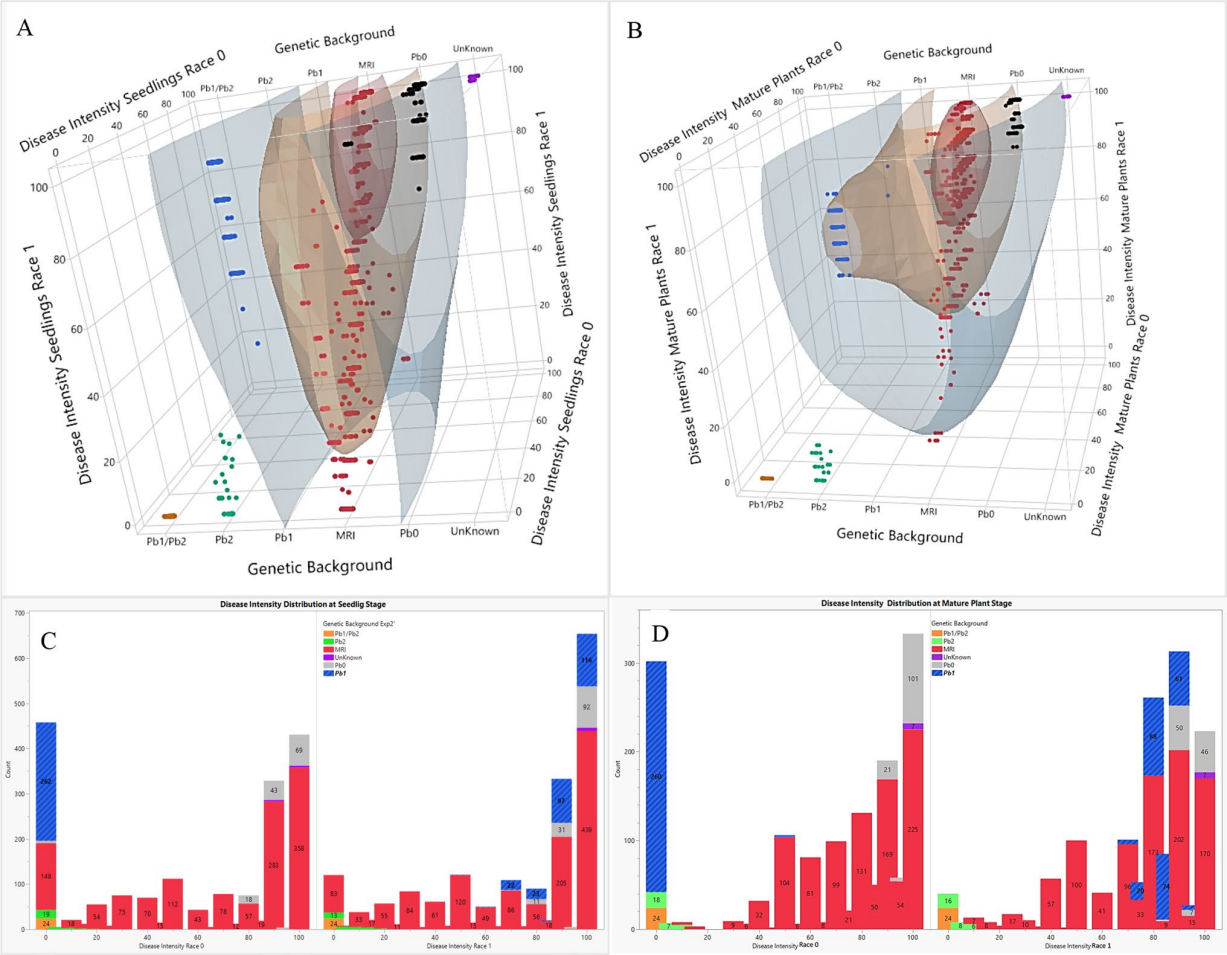
**A-B** 3D-scatter plots of the disease intensity (D.I) of the genetic backgrounds at seedling or mature stages inoculated by different isolates. **C**-**D** The D.I distribution histograms of seedlings or mature plants by different isolates. **A** and **C** *n* = 1851; **B** and **D** *n* = 1279

Correlation data are visualized using a 3D-scatter plot. The plot represents the correlation between D.I at the seedling stage and the adult stage for race 0 and race 1 (Fig. 7 A, B). The histograms represent the distribution of plants in each experiment used for the correlation analysis (Fig. 7 C, D). The three-dimensional scatter plot shows plant dispersion of all genetic backgrounds. Quantile analysis was used to compare the dispersion of the genetic backgrounds. The quantile represents the similarity of resistance between the genetic backgrounds using specific outliers. The outlier is considered any value more than Q times the interquartile range from the lower and upper quantiles. Out of the gray area is the upper decile (0.1), in orange the median (0.5), and in red the lower decile (0.9). The decile outliers are the same for Figs. 7 and 8.

In the upper decile of Fig. 7A, one can see the *Pb1/Pb2* and unknown ('Eleonora') background, while between the upper and middle decile, one can see *Pb2*, *Pb1* and several plants from the MRI and *Pb0* background. *Pb1/Pb2* (orange color) were highly correlated with low values (0) for both races, while 'Eleonora' D.I was strongly correlated with high values (100) for both races (purple color). The other genetic backgrounds were found in the median or lower decile. The dispersion trend appears to be the same for both growth stages. When D.I was examined in both growth stages that were subjected to race 1, the *Pb1/Pb2* and *Pb2* genetic backgrounds belonged to the upper decile, *Pb0* and part of the MRI belonged to the sixth-to-ninth decile, while the other genetic backgrounds including *Pb1* belonged to the median or lower decile (Fig. 7B). The distribution of the genetic backgrounds shown by the histogram between the seedling stage and the adult stage under race 0 indicates a high similarity with minor changes. The histograms of Fig. 7C and D show the D.I distrubution of seedlings and adult plants exposed to race 0 and race 1. For both races, D.I distribution between seedling and adult plants stayed consistant (Fig. 7C, Experiments 1 and 3 for race 0 and Fig. 7D, Experiments 2 and 4 for race 1). When the correlation was examined by growth stage for the different races, the correlation of the *Pb* genetic backgrounds was low (0.31) for seedling and adult plants (0.4). These low values mainly indicate the effect of the race used. In contrast, when the correlation was examined by growth stage for the different races, the correlation of the MRI resistance background was high for seedlings (0.95) and for adult plants (0.73). These high correlation values indicate that the dependence of MRI background on age and race is weaker than that of *Pb* backgrounds (see also Table 4). In the upper decile of Fig. 8A, we see *Pb1/Pb2, Pb2*, a number of individual MRI-derived plants and 'Eleonora', while between the upper and middle decile, we see *Pb1* and few plants from the MRI and *Pb0* background. All others were found in the median or lower decile. When D.I was examined in adult stage and subjected to the two races, the genetic backgrounds *Pb1/Pb2* and *Pb2* belonged again to the upper decile, while *Pb0* and part of the MRI belonged to the sixth-to-ninth deciles, while the other genetic backgrounds including *Pb1* belonged to the median or lower decile (Fig. 8B). The D.I dispersion histograms trend appears to be the same in both growth phases (Fig. 8C and D).

When the correlation was examined by combining race and age together, the correlation of the *Pb* background was low (0.38 and 0.31), while that of MRI background increased to 0.53 and 0.55 (Table 4 last row). These results together with the distribution diagram show that the *Pb* gene group is more dependent on race/age combination, unlike the MRI-group which shows reduced dependency on race and age.

### Regression and prediction

To better understand the contribution and strength of each component to D.I and to gain an ability to predict its development under all variables, we conducted a series of regression analyses based on an actual model as well as expected models. First, we analyzed Experiments 1–4 to obtain the RMSE, *R*^*2*^ values, and regression equation. The environmental factors used were mean minimal temperature (Tm), mean maximal Tm, mean Dew Point, minimum RH, and number of days (by %) with more than 6 h > 95%RH.

We reduced the number of variables from dozens to a minimum by maintaining low RMSE and high *R*^*2*^ values, keeping variables that may aid growers and breeders to identify resistant plants at early stages of disease development. We found that the daily DDR and sporulation rate had the strongest effect on prediction of D.I.

We then used Experiments 5–6 to test the strength of the prediction model (Experiment 5, plants were inoculated first with race 0 and 10 days later with race 1; Experiment 6, unidentified race of *P. belbahrii* in NJ). Since marketing rate is an ordinal and not continuous variable, we used the Ordinal Logistic Fit model. The analysis included 8071 plants and 13 degree of freedom. An *R*^*2*^ of 0.85 was obtained with Akaike information criterion (AIC) of 2259 and Bayesian information criterion (BIC) of 2317 (Table 5).

**Table 5:** Ordinal logistic fit: whole model test

Model	-LogLikelihood	DF	Chi-square	*P* > ChiSq
Difference	6464.0471	13	12,928.09	*P* <.0001
Full	1113.6713			
Reduced	7577.7185			

RSquare (U)	0.8530
AICc	2259.41
BIC	2371.28
Observations	8071

### Prediction by actual model

We chose the D.I index for prediction due to its high correlation with the marketing rate (*R* = 0.957). To predict D.I at the end of the epidemics, we incorporated into the model all scoring data (5–6 scores per Experiment) and all measured environmental factors. We first fit all 29 variables of all Experiments into the model (3 indices, 6 score times, AUDPC, SAUDPC, DDR, growth stage, genetic background, race, av. min Tm, av. max Tm, av. Dew Point, Av. night RH, and % of days 6 h ≥ RH 95%). Then, we removed the values whose influence on the model was extremely minor. It came out that the model can predict the final D.I using three main variables (sporulation rate, DDR, and plant age) with *R*^*2*^ = 0.93 and RMSE = 9.34 for Experiments 1–4, *R*^*2*^ = 0.93 and RMSE = 8.71 for Experiments 5–6, and *R*^*2*^ = 0.91 and RMSE = 10.56 for all experiments (Table 6). The similar *R*^*2*^ and RMSE values that were obtained for all experiments, both separately and together, testify for a strong prediction model that meets the differences in genetics, races, age, and environments. To enable the grower or breeder to predict the intensity of the disease at an early stage of the epidemics and to perform an effective disease management or selection, we tested the model using the above indices at an early stage of the epidemics. The model predicted D.I at the second score with *R*^*2*^ = 0.85 and RMSE = 13.9 for Experiments 1–4, *R*^*2*^ = 0.81 and RMSE = 13.7 for Experiments 5–6, and *R*^*2*^ = 0.82 and RMSE = 15.03 for all Experiments 1–6 (Table 6). Again, similar *R*^*2*^ and RMSE values for all experiments, separately and together, indicate a strong prediction model that responds to different races, genetics, age, and environments. The prediction of the disease based on the sporulation in the second scoring reached *R*^*2*^ = 0.82 as against a maximal *R*^*2*^ = 0.91 in the last score, thus providing 90% accuracy in prediction (Table 6).

**Table 6:** Summary of fit and analysis of variance

		Summary of fit					Analysis of variance			
	Experiments	*R*^*2*^	*R*^*2*^ (Adj)	RMSE	MR	Ob	DF	SS (total)	MS	F ratio*
Sporulation last score	1–4	0.93	0.93	9.34	66.7	6,528	10	7,650,412	765,041	8764.9
	5–6	0.93	0.93	8.71	68.9	1,543	8	1,456,963	182,120	2397.9
	1–6	0.91	0.91	10.56	67.1	8,071	10	8,900,425	890,043	7980.1
Sporulation 2nd score (out of 5)	1–4	0.85	0.85	13.9	66.8	6,562	10	6,979,065	697,907	3,610
	5–6	0.82	0.81	13.7	68.9	1,548	8	1,283,993	160,499	847.3
	1–6	0.82	0.82	15.03	67.2	8,110	12	9,826,701	666,523	2951.6

*MR* Mean Response,* DF* Degree of Freedom, *SS* Sum of Squares, *MS* Mean of Square

^*^P > 0.001

**Fig. 9:**
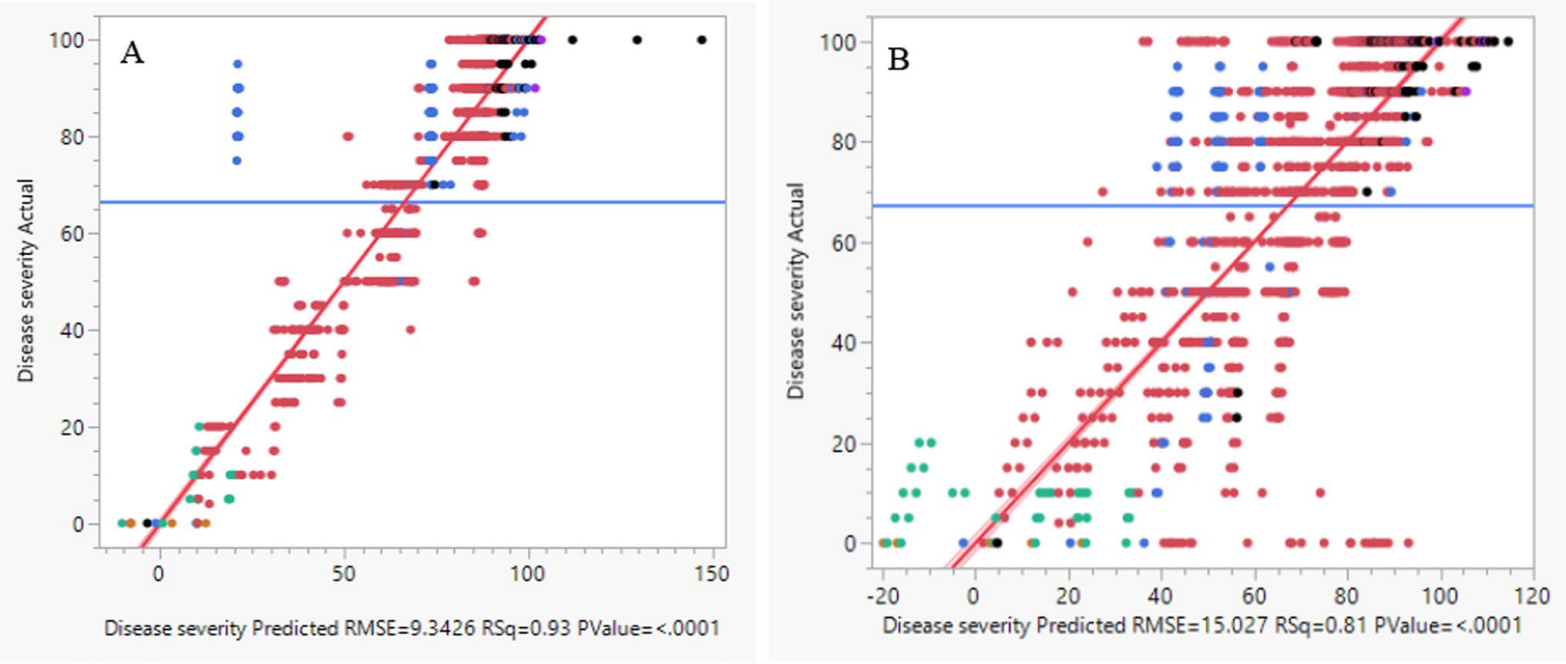
**A** The maximal prediction of disease severity based on disease development rate (DDR) and sporulation at the last score rating, *R*^*2*^ = 0.93. **B** Prediction model of disease severity based on DDR and sporulation at the second score rating (~ 2/5 time of the experiment) was *R*^*2*^ = 0.81 (*P* ≤ 0.0001)

Figure 9A shows the minimal dispersion of the plants around the predication trend line in the last score of D.I (*R*^*2*^ = 0.93, RMSE = 9.34) which represents the maximal prediction ability of the model, while Fig. 9B shows a wider dispersion in the 2nd score (*R*^*2*^ = 0.81, RMSE = 15.03). This time of recording represents the first timing when all inoculated plants align with the prediction trend line and correctly reflect the future D.I of the plants. In the 1 st score, the prediction was low (*R*^*2*^ = 0.59, RMSE = 23.7), while in the 3rd score and the 4 th score, the *R*^*2*^ and RMSE were higher, and similar but slightly lower than the last record (~ *R*^*2*^ = 0.91, RMSE = 9.45 and ~ *R*^*2*^ = 0.91, RMSE = 9.4, respectively).

Using Jmp Profiler, we found improved selection was achieved at the seedling stage than the mature stage. Choosing plants without any level of sporulation and a daily development rate of the disease is less than 2.9% per day. The required conditions are that 39–95% of the days should be with 6 h of RH > 95% at Tm of 19–23.5 °C.

The regression and prediction formulae of D.I were done with Jmp fit least square prediction formula script. To reduce the data used in the equation and develop a uniform model for all plants, we reduced all the genetic backgrounds to one genetic identity. Model 1 is a prediction model based on all six experiments according to the intensity of sporulation in the last scoring, while model 2 shows the prediction ability based on the intensity of sporulation in the second scoring.

Model 1. The Pred Formula D.I of trial 1–6 at the last scoring:

$$\begin{gathered} \, = \,59.0713188736328\, + \, - 0.981612236266427{\text{ }}*{\text{ }}\% {\text{ }}6~h - RH95n\, + \,4.16443532367042{\text{ }}*{\text{ }}Daily{\text{ }} \hfill \\ Rate\, + \,18.0564078491357{\text{ }}*{\text{ }}Sporulation\, + \,1.32618616341241{\text{ }}*{\text{ }}Tm{\text{ }}\max \, + \,Match{\text{ }} \hfill \\ \left( {Growth{\text{ }}Stage,{\text{ }}Cotyledon,{\text{ }}0,{\text{ }}Field,{\text{ }}0,} \right)\, + \,Match{\text{ }}\left( {Race,{\text{ }}Race{\text{ }}0,{\text{ }}0,Race{\text{ }}1,{\text{ }}0.0440960524256607,{\text{ }}Race{\text{ }}1\, + \,2, - 25.6999841512866,{\text{ }}Undefined, - 21.4975434012421} \right) \hfill \\ \end{gathered}$$

Model 2. The Pred Formula Disease intensity of trial 1–6 at the 2nd score:

$$\begin{gathered} \, = \,\left( { - 112.910303157038} \right)\, + \,8.42518792211718{\text{ }}*{\text{ }}Daily{\text{ }}Rate\, + \,Match{\text{ }}\left( {:Growth{\text{ }}Stage,{\text{ }}Cotyledon,{\text{ }}0,{\text{ }}Field,{\text{ }}34.3795974669999} \right) \hfill \\ \, + \,4.63083180089536{\text{ }}*:Tm{\text{ }}\max \, + \,0{\text{ }}*:\% {\text{ }}6~h - RH95n\, + \,10.3360506451271{\text{ }}*:Sporulation{\text{ }}\text{Re} ad{\text{ }}2)\, \hfill \\ + \,Match\left( {:Race,{\text{ }}Race{\text{ }}0,{\text{ }}0,{\text{ }}Race{\text{ }}1,{\text{ }}2.22474519861588,{\text{ }}Race{\text{ }}1\, + \,2, - 9.64739315719014,{\text{ }}Undefined,{\text{ }}26.8965232457966} \right) \hfill \\ \end{gathered}$$

## Discussion

We conducted six experiments to examine the ability of 150 *Ocimum basilicum* accessions to resist BDM infection based on their genetic background, age, pathogen race, and environmental conditions. *Pb1/Pb2* background supplied plants with high resistance, independent of race 0, 1, or 0 + 1 at all growth stages and environmental condition. *Pb2* plants showed independence of race and environment with minor dependence on age. *Pb1* plants showed full dependence on race which ranged from 0% disease for race 0 to almost 100% disease for race 1 at seedling and adult growth stage. The highest variable response to disease was the MRI cultivars and their RILs. However, partition of all MRI cultivars and their RILs into minimal groups showed that several RILs had a high resistance to BDM, similarly to the *Pb2* genetic background. *Pb0* cultivars and'Eleonora'(unknown background) were susceptible to all races in all growth stages in all experiments. Excluding the specificity of the genetic background (*Pb1*) and considering the highly resistant group from MRI-RIL's partitioning, a general ranking of all genetic backgrounds is as follows: *Pb1/Pb2* > *Pb2* > MRI > *Pb1* > *Pb0* and *Unknown.*

A wide-range examination that included all genetic background showed good correlation (R = 0.73) between D.I at seedling stage under controlled conditions and D.I of adult plants under field conditions. For all six Experiments, PCA analysis showed that 1 st component strength of 65% was affected by the genetic background the 2nd component strength of 22% were affected by the race and the 3rd component of 7% and was affected by the growth stage. The resistance prediction model was evaluated based on two statistical indices, residual (%) and root-mean-square error (RMSE). The best predictability was based on the sporulation and DDR indices. Based on these indices, the resistance prediction probability at the second score (~ 2/5 time of the experiment) rating was *R*^*2*^ = 0.82, while at the third scoring (~ 1/2 time the experiments) and the last scoring, the *R*^*2*^ was 0.91 and 0.93, respectively.

Using the JMP prediction Profiler, we adjusted the set of variables with the highest probability for selecting a resistant plant with commercial rating of 1 for all genetics backgrounds and races simultaneously. It was found that selecting individual resistant plants should be better done at the seedling stage. Selected plants should show (i) no or slight sporulation (score 1) and (ii) average daily development rate of < 3% per day. In the field, the best selection should be made when 6 h of RH > 95% with Tm of 23.5 °C prevails for at least 45% of the days in an experiment. Slight positive deviation in sporulation and DDR decreases the number susceptible plants of *Pb1* and MRI-RIL's compared with *Pb2* and *Pb1/Pb2*. Moreover, the prediction model indicates that the commercial grade (Marketing Rate1) is highly reflected by the sporulation. The incidence of plants showing even the slightest sporulation at an early stage was ranked as nonmarketable later. At this current time, introgression of dominant resistance genes via interspecific hybridization appears to be the most successful approach to ensure “complete” resistance.

Commercial BDM-resistant cultivars are currently available. They do provide significant relief to farmers and, in many cases, have saved their crops. Yet, the available BDM-resistant cultivars may behave differentially under different biotic and abiotic environments. The model we developed can predict the final D.I of an entry based on fungal sporulation and daily rate of disease development at an early stage of the epidemics, thus facilitating more timely management decisions for breeders and growers. The resistant cultivars are based on several different genetic backgrounds, which can allow for more flexible responses that provide more options to growers and permit the plant breeders to incorporate a wider range of genetic backgrounds to meet the changing dynamics of the pathogen. Growers can try their own selection of cultivars with varying disease resistance backgrounds in their fields and adjust according to their cultivation method, growth stage, climate conditions, and the prevalence of existing races ultimately by assessing the response of the particular basil plants being evaluated. Currently, the richness of the disease resistant cultivars available is limited and more are needed. However, the use of good farming practices along with an integrated strategy of disease control products correctly timed and applied can further protect the basil crop even when growing resistant basils (Cohen et al. 2017). The dynamic pathogenicity of *P. belbahrii* is not clear or fully understood, but given the downy mildew races found in many other crop species, we should anticipate that new races will emerge and that new sources of resistance will be needed.

## Data Availability

Data will be made available on reasonable request.

## Abbreviations

AUDPC: Area under disease progress curve
BDM: Basil downy mildew
DDR: Disease development rate
D.I: Disease intensity
M.R: Marketing rating
MRI: ‘Mrihani’ (RU Collection)
RH: Relative humidity
RIL’s: Recombinant inbred lines
RMSE: Root-mean-square error
Tm: Temperature
